# Immature Platelet Fraction as a Surrogate Marker of Thrombo-Inflammation in Hospitalized COVID-19 Patients

**DOI:** 10.3390/life15121846

**Published:** 2025-11-30

**Authors:** Adrian Duek, Alexandra Zimin, Yael Hershkop, Michal Cipok, Amir Cohen, Merav Leiba

**Affiliations:** 1 Hematology Institute, Samson Assuta Ashdod University Hospital, Ashdod 7747629, Israel; meravlei@assuta.co.il; 2 Faculty of Health Sciences, Ben-Gurion University of the Negev, Beer Sheva 8410501, Israel; 3 Pathology Institute, Samson Assuta Ashdod University Hospital, Ashdod 7747629, Israel; 4 Laboratory Division, Samson Assuta Ashdod University Hospital, Ashdod 7747629, Israel; 5 Cardiology Department, Samson Assuta Ashdod University Hospital, Ashdod 7747629, Israel

**Keywords:** COVID 19, inflammation, immature platelet fraction, white blood cell count

## Abstract

Although COVID-19 is associated with significant thrombo-inflammatory complications, reliable biomarkers to guide antithrombotic therapy remain limited. Immature platelet fraction (IPF) reflects platelet turnover and may indicate heightened thrombotic risk. We retrospectively analyzed 133 hospitalized COVID-19 patients (median age 68 years) at a single center. IPF and inflammatory markers (WBC, ANC, D-dimer, LDH, CRP) were measured on admission. Correlations between IPF and these biomarkers were assessed overall and in clinical subgroups (age, sex, disease severity, comorbidities, and treatment). We found that IPF was positively correlated with WBC and ANC in patients less than 70 years old (r = 0.36 and 0.33, respectively; *p* < 0.05), males, and those with moderate-to-severe disease. Among patients with congestive heart failure, IPF correlated strongly with D-dimer (r = 0.78, *p* = 0.013). Similar associations were observed in patients requiring enoxaparin or antiplatelet therapy. No significant correlations were found in patients age 70 or older. Based on these findings, we conclude that elevated IPF is associated with increased inflammatory and thrombotic activity in hospitalized COVID-19 patients, especially in younger, male, and more severe cases. These findings suggest IPF may serve as a dynamic marker for thrombo-inflammation and help identify patients who might benefit from more intensive antithrombotic therapy. Larger studies are warranted to validate IPF as a biomarker for personalized management of COVID-19.

## 1. Introduction

Over the last two decades, coronavirus disease has emerged as a global health threat due to its accelerated geographic spread. The origin of the virus has a zoonotic source. Human-to-human transmission occurs through direct contact and airborne routes [1]. The Coronavirus Disease of 2019 (COVID-19) was caused by the coronavirus Severe Acute Respiratory Syndrome Coronavirus 2 (SARS-CoV-2).

In addition to respiratory illness, this virus increases the risk of venous and arterial thrombotic events. SARS-CoV-2 infection can cause microvascular thrombosis as a result of endothelial dysfunction in the pulmonary vascular bed and possibly in other vessels [2].

The risk of thrombotic complications in COVID-19 cases has been associated with various biomarkers related to inflammation, such as C-reactive protein (CRP), ferritin, D-dimer, lactate dehydrogenase (LDH), platelet-to-lymphocyte ratio (PLR), and neutrophil-to-lymphocyte ratio (NLR). Other inflammation biomarkers described include alanine aminotransferase (ALT) activity and aspartate aminotransferase (AST) activity [3].

While established biomarkers such as NLR, PLR, and D-dimer have proven useful in the risk stratification of COVID-19 patients, they have important limitations. D-dimer reflects fibrin degradation but lacks specificity for active thrombotic risk, as it is elevated in numerous inflammatory conditions. NLR and PLR are calculated ratios that reflect systemic inflammation but do not directly assess platelet activation or turnover. In contrast, immature platelet fraction (IPF) provides a direct, real-time measure of thrombopoiesis and platelet turnover, reflecting the bone marrow’s response to consumptive or inflammatory stimuli.

Patients with severe COVID-19 exhibit a prothrombotic state, with their platelets displaying a hyperactive, procoagulant phenotype typical of young, immature platelets [4]. The incidence of thrombotic events remains elevated up to 48 weeks after a COVID-19 diagnosis [5]. Studies have shown that patients with COVID-19 have more immature platelets, defined as immature platelet fraction (IPF), compared to those with other conditions known for high levels of immature platelets [6]. Additionally, there is an association between IPF indices and the severity of COVID-19 disease [7].

Immature platelets are larger, more reactive, and possess greater thrombotic potential than mature platelets, making IPF a dynamic marker of ongoing platelet activation. Furthermore, IPF can be measured rapidly using routine automated hematology analyzers without additional cost or specialized testing, thus offering a practical advantage in clinical settings. Therefore, IPF may serve as a complementary biomarker that integrates information about both inflammatory drive and prothrombotic risk, potentially identifying patients who would benefit from intensified antithrombotic therapy.

The IPF or reticulated platelets (RP) are newly released thrombocytes. They differ from mature platelets in their larger size and higher concentration of RNA in the cytoplasm, as well as by their early release into circulation from the bone marrow via megakaryocytes [8]. There is a strong correlation between the levels of IPF and RP in the blood [9,10]. These platelets represent a population of hyper-reactive platelets associated with thrombotic propensity and have a greater tendency for thrombus formation [6]. IPF measures the proportion of immature platelets in the total platelet count in peripheral blood. The IPF is measured using automated analyzers that utilize flow cytometry techniques and RNA-specific dyes to quantify these immature platelets [11,12].

Additionally, IPF can be used to predict platelet recovery after chemotherapy and successful engraftment following stem cell transplantation [13]. It has also shown utility in other clinical scenarios, including acute coronary syndromes, where increased IPF levels may indicate heightened thrombotic activity [10]. The IPF is closely related to inflammation and can serve as a biomarker for various inflammatory conditions. Elevated IPF levels have been observed in patients with sepsis, where they correlate with disease severity and prognosis. In critically ill patients, higher IPF levels can predict the onset of sepsis before clinical symptoms become apparent [14,15,16].

The current study evaluated the correlation among IPF levels and thrombosis-related inflammatory biomarkers in patients with COVID-19. Additionally, we investigated the incidence of thrombosis in this population within 48 weeks after COVID-19 diagnosis.

## 2. Materials and Methods

This was a retrospective, cross-sectional study of patients diagnosed with COVID-19 infection and hospitalized at Assuta Ashdod University Hospital, Israel.

Patients aged 18 years or older with SARS-CoV-2 infection who were admitted to Assuta Ashdod University Hospital from 30 March to 28 April 2020 were included. Additional inclusion criteria were at least one positive reverse transcription polymerase chain reaction (RT-PCR) test for SARS-CoV-2 infection, patients for whom IPF levels were determined at admission, and patients for whom complete blood count, D-dimer, ferritin, ALT, AST, LDH, and CRP levels were determined at admission.

Exclusion criteria were admission to the hospital for a different condition, patients with an additional significant illness at admission, those with active inflammatory disease, those with active cancer diagnosis, those undergoing immunosuppressive treatment, or those for whom no IPF test was performed.

### 2.1. Study Protocol

IPF was measured using 3 mL of whole blood collected in EDTA tubes. The inflammation index tests were based on a 5 mL blood sample that was drawn into a lithium heparin tube. Erythrocyte sedimentation rate was tested using a 1 mL blood sample drawn into a citrate tube. COVID-19 was diagnosed based on one or more positive RT-PCR (real-time PCR) tests. COVID-19 disease severity was classified according to the COVID-19 Treatment Guidelines published by the National Institutes of Health. Patients were categorized as follows: (1) mild disease: individuals with symptoms but without shortness of breath, dyspnea, or abnormal chest imaging; (2) moderate disease: evidence of lower respiratory disease with oxygen saturation (SpO_2_) ≥ 94% on room air; (3) severe disease: SpO_2_ < 94% on room air, ratio of arterial partial pressure of oxygen to fraction of inspired oxygen (PaO_2_/FiO_2_) < 300 mmHg, respiratory rate > 30 breaths/min, or lung infiltrates > 50%. For analytical purposes, patients with mild and moderate disease were grouped together and compared with those with severe disease, as the latter group was more likely to exhibit significant thrombo-inflammatory complications [17]. IPF levels and other inflammation markers (CRP, ferritin, LDH, D-dimer, NLR, PLR, AST, and ALT) were measured in peripheral blood samples.

The IPF from the blood samples was evaluated using an automated Sysmex XN-3000 analyzer (Sysmex America, Inc., Mundelein, IL, USA), which uses fluorescent dyes containing oxazine and ethylene glycol. This system can discriminate between mature and immature platelets and report the immature platelet fraction as a percentage.

Results for the following parameters were extracted from the patients’ electronic medical records: (1) demographics (age, sex, weight, previous medical history, concomitant medications); (2) laboratory results: complete blood counts and chemistry, LDH, ferritin, CRP, D-Dimer, platelet to lymphocyte ratio, NLR, ALT, AST; and (3) COVID-19 severity defined according to the COVID-19 Treatment Guidelines [18].

### 2.2. Statistical Analysis

Categorical variables are reported as frequencies and percentages, while the distribution of continuous variables was evaluated using a histogram and the Kolmogorov–Smirnov test. Continuous variables that were normally distributed are described as mean and standard deviation, while others are reported as median and interquartile range (IQR).

The correlation among biomarkers was evaluated using the Pearson correlation coefficient, depending on the distribution of the variables. To address the potential confounding effects of continuous variables, partial correlation was used. To control for categorical confounders, the dataset was stratified by category, and the correlation coefficient was calculated separately for each stratum (for example, for males and females separately). The correlation coefficients were compared among the categories using Fisher Z Transformation.

Considering multiple comparisons, *p*-values were adjusted using the False Discovery Rate (FDR)—Benjamini–Hochberg Procedure. This adjustment reduces the risk of Type I (false positive) errors.

All patients included in the final analysis had complete data for IPF and the primary inflammatory markers (WBC, ANC, CRP, D-dimer, and LDH) at admission. Patients with missing IPF values or incomplete baseline laboratory assessments were excluded during the screening phase. No imputation methods were used, as the proportion of missing data was minimal (<5%) and observed to occur randomly.

The sample size of 133 patients was determined by the availability of consecutive hospitalized COVID-19 patients during the study period (30 March to 28 April 2020) who met all inclusion criteria. Post hoc power analysis indicated that, with *n* = 133, we had 80% power to detect a correlation coefficient of r ≥ 0.24 at α = 0.05 (two-tailed). For subgroup analyses, we calculated the required sample size to achieve adequate power for observed correlations and reported these values in Table 2 when statistical significance was not reached (*n* req.). Correlations were considered robust when the observed sample size exceeded the calculated minimum required sample size and the adjusted *p*-value was <0.05.

All statistical analyses were two-sided, and significance was defined as a *p*-value < 0.05.

## 3. Results

### 3.1. Baseline Characteristics

A total of 133 consecutive patients were included in this study. The median age of the entire population was 68 years (range 19–101). Forty-seven percent (*n* = 62) were older than 70 years. Eighty-four patients (63%) were male, and the median weight was 83.5 kg (range: 50–130 kg). Fifty-three patients (40.5%) had mild or moderate disease, while seventy-eight (59.2%) presented with severe disease. Eight patients (6%) initially had mild disease, which deteriorated to severe disease. The mean duration of hospitalization was 9.1  ±  5.5 days.

Regarding co-morbidities known to be risk-factors for thrombosis, 11% of the population were smokers (*n* = 13), 17% had ischemic heart disease (*n* = 23), 43% had diabetes (*n* = 57), and 44% had hyperlipidemia (*n* = 58). Additionally, 13% of the patients had chronic renal failure (*n* = 17), and 13.5% (*n* = 18) were diagnosed with chronic respiratory diseases, such as COPD or asthma (Table 1).

The laboratory parameters associated with thrombosis risk at study entry, along with the baseline characteristics of the patients, are also summarized in Table 1.

The IPF demonstrates variable correlations with inflammatory and thrombotic markers in COVID-19 patients, with the significance of these correlations often being dependent on subgroup analyses. While some correlations were observed, many lost significance after adjustments for multiple comparisons, and the required sample sizes for drawing robust conclusions were often not achieved (Table 2). By focusing on subgroups with larger sample sizes and more consistent statistically significant findings, we identified a regular trend across multiple subgroups of a correlation between IPF, white blood cell (WBC) count, and absolute neutrophil count (ANC). The median IPF for the total cohort was 1.9% (IQR 0.2−25, range 0.1−29). The median IPF values for key clinical subgroups are presented in Table 3.

**Figure 1:**
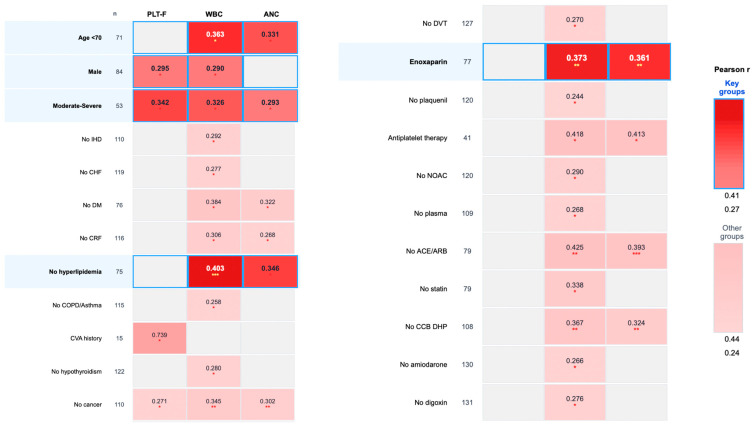
IPF Correlations with platelet and myeloid markers across clinical subgroups. Color intensity represents Pearson correlation coefficient (r) values, with red indicating positive correlations. Highlighted rows represent key subgroups with robust findings (darker, saturated colors). Other rows show additional correlations with lighter shading. Only subgroups with n ≥ 50 and adjusted *p* < 0.05 are shown. Asterisks denote statistical significance: * *p* < 0.05, ** *p* < 0.01, *** *p* < 0.001. Abbreviations: PLT-F: fluorescence platelet count; WBC: white blood cell count; ANC: absolute neutrophil count; IHD: ischemic heart disease; CHF: congestive heart failure; DM: diabetes mellitus; CRF: chronic renal failure; DVT: deep vein thrombosis; NOAC: new oral anticoagulants; ACE/ARB: ACE inhibitors/ARB; CCB DHP: dihydropyridine calcium channel blocker.

The most consistent and statistically robust correlations were observed between IPF and markers of myeloid activation (WBC and ANC). These associations were particularly strong in the following subgroups:

- Age < 70 years (*n* = 71): IPF vs. WBC (r = 0.363, *p* = 0.026) and ANC (r = 0.331, *p* = 0.033).
- Male patients (*n* = 84): IPF vs. WBC (r = 0.290, *p* = 0.046) and PLT-F (r = 0.295, *p* = 0.046).
- Moderate-to-severe disease (*n* = 53): IPF vs. PLT-F (r = 0.342, *p* = 0.026), WBC (r = 0.326, *p* = 0.026), and ANC (r = 0.293, *p* = 0.039).
- Patients without hyperlipidemia (*n* = 75): IPF vs. WBC (r = 0.403, *p* = 0.0004) and ANC (r = 0.346, *p* = 0.013).
- Patients requiring enoxaparin (*n* = 77): IPF vs. WBC (r = 0.373, *p* = 0.007) and ANC (r = 0.361, *p* = 0.007); see Figure 1.

Notably, in the small cohort of patients with congestive heart failure (*n* = 14), IPF showed a strong correlation with D-dimer (r = 0.785, *p* = 0.013), suggesting heightened thrombotic risk in this vulnerable population.

### 3.2. Comorbidities

The small cohort of patients with congestive heart failure (*n* = 14) was the only subgroup in which IPF showed a strong correlation with D-dimer (r = 0.78, adjusted *p* = 0.013).

Among patients without diabetes mellitus (*n* = 76), IPF was positively correlated with WBC (r = 0.384, adjusted *p* = 0.013) and ANC (r = 0.322, adjusted *p* = 0.033). Notably, in the 24 individuals with non-insulin-dependent diabetes mellitus, an inverse relationship between lymphocytes and the NLR was observed (r = −0.438, *p* = 0.260), possibly reflecting immune dysregulation.

IPF was significantly correlated with WBC (r = 0.306, adjusted *p* = 0.013) and ANC (r = 0.268, adjusted *p* = 0.026) in patients with normal renal function (*n* = 116).

Among subjects with a normal lipid profile (*n* = 75), IPF exhibited significant correlations with WBC (r = 0.403, adjusted *p* = 0.00039) and neutrophils (NEU, r = 0.346, adjusted *p* = 0.013).

A strong association between IPF and PLTF (r = 0.739, adjusted *p* = 0.026) was found among patients with a history of cerebrovascular accident (*n* = 15).

Analysis based on cancer history revealed notable differences: in patients without a history of cancer (*n* = 110), IPF correlated positively with PLTF (r = 0.271, adjusted *p* = 0.013), fibrinogen (r = 0.361, adjusted *p* = 0.009), WBC (r = 0.354, adjusted *p* = 0.0026), and NEU (r = 0.302, adjusted *p* = 0.007). However, no significant correlations were observed in patients with a history of cancer (*n* = 23). The relatively small sample size may limit the statistical power.

### 3.3. Treatment-Related Associations

Seventy-seven participants required enoxaparin. IPF correlated significantly with WBC (r = 0.373, adjusted *p* = 0.007) and NEU (r = 0.361, adjusted *p* = 0.007). In patients requiring antiplatelet therapy (*n* = 41), IPF was significantly correlated with WBC (r = 0.418, adjusted *p* = 0.046) and LDH (r = 0.412, adjusted *p* = 0.046).

Among those treated with plaquenil (*n* = 13), IPF showed a strong correlation with LDH (r = 0.843, adjusted *p* = 0.013). The significance of this finding in such a small population—and whether it reflects a beneficial or adverse effect of plaquenil on platelet activation and tissue damage—requires further investigation.

## 4. Discussion

This study investigated the relationship between IPF and various inflammatory and thrombotic markers in patients hospitalized with COVID-19, considering the influence of age, sex, disease severity, comorbidities, and treatments.

We acknowledge that the Pearson correlation coefficients (r) observed between IPF and inflammatory markers are generally modest, suggesting that only a fraction of the variance is shared. For example, the strongest correlation (r = 0.403 for IPF vs. WBC in patients without hyperlipidemia) translates to an r^2^ of approximately 16%, indicating that IPF explains only 16% of the variance in WBC in this subgroup. However, in the context of complex, multifactorial biological processes like COVID-19 thrombo-inflammation, correlations of this magnitude, which are statistically significant after adjustment for multiple comparisons, are biologically meaningful. They support a coordinated pathophysiological link rather than direct causation, emphasizing IPF’s role as an adjunctive marker that integrates thrombotic and inflammatory information.

The positive correlations observed between IPF and both WBC and ANC in COVID-19 patients—particularly among those under 70 years of age, male patients, and individuals with moderate-to-severe disease—suggest a coordinated hematopoietic response to SARS-CoV-2-induced inflammation. This pattern aligns with published evidence of emergency myelopoiesis in severe COVID-19, where systemic inflammation drives accelerated production and release of immature myeloid cells (including immature platelets) from bone marrow. SARS-CoV-2 infection alters hematopoietic stem cell differentiation, favoring myeloid lineage expansion over lymphoid production—a hallmark of emergency myelopoiesis observed in severe cases.

COVID-19 is characterized by a hyperinflammatory state that could drive both increased platelet turnover and elevated WBC counts [19]. It has been demonstrated that inflammatory cytokines, such as IL-6 and TNF-α, stimulate thrombopoiesis [20,21,22]. These cells exhibit pro-thrombotic and hyperinflammatory phenotypes, perpetuating endothelial injury [23]. This finding reinforces the concept that IPF is not a causative agent of inflammation but rather a dynamic biomarker reflecting the coordinated hematopoietic response to the SARS-CoV-2 induced hyper-inflammatory state. The increased release of immature platelets, which are known to be hyper-reactive and procoagulant, is part of this overall pro-thrombotic milieu. Therefore, elevated IPF serves as a practical, accessible measure of the heightened thrombotic risk driven by the systemic inflammation. Furthermore, we acknowledge that mean platelet volume (MPV) is often considered an indirect marker of young platelets. However, we opted to focus solely on IPF because it is a direct and specific measure of thrombopoiesis, utilizing RNA-specific dyes via flow cytometry techniques, which offers greater accuracy and clinical relevance for platelet turnover compared to the less specific measure of MPV. Multiple studies on myeloproliferative neoplasms, including large cohort analyses and meta-analyses, have also established the role of increased myelopoiesis, particularly leukocytes and platelets, as independent risk factors for both arterial and venous thrombotic events [24,25,26].

In addition, we found that patients requiring enoxaparin had higher IPF and WBC/NEU levels. These results could reflect the severity of the disease or may indicate ongoing inflammation despite anticoagulation, suggesting that these individuals may need higher doses or alternative anticoagulation strategies.

Moreover, IPF positively correlates with WBC in patients on antiplatelet therapy, suggesting that platelet activation and inflammatory responses might require further suppression in these individuals. This finding underscores the importance of the careful monitoring and potential intensification of antithrombotic strategies in high-risk COVID-19 patients receiving antiplatelet agents.

The significant correlation between IPF and WBC/ANC in patients under 70 years of age, accompanied by the absence of these correlations in older patients, suggests age-related differences in the inflammatory and thrombopoietic responses to COVID-19. Younger individuals may mount a more robust inflammatory response, leading to greater stimulation of platelet production.

We also found a correlation between IPF and comorbidities, especially with congestive heart failure. The observation that IPF correlated with D-dimer in patients with congestive heart failure highlights the potential for increased thrombotic risk in this vulnerable population. CHF is a known risk factor for thromboembolism [27], and COVID-19 may further exacerbate this risk through increased platelet activation and coagulation. However, the small sample size in this subgroup warrants cautious interpretation.

The interplay between diabetes and COVID-19 appears more complex. The direct correlation we observed between IPF and lymphocyte count may reflect a distinct immune-hematological response in this subgroup. In diabetes, chronic low-grade inflammation and immune dysregulation are well-documented [28] often resulting in reduced lymphocyte function and altered leukocyte profiles. However, during acute viral infection such as COVID-19, some patients may mount a relatively preserved or even compensatory lymphocyte response, particularly if their diabetes is well-controlled or they are less severely affected by hyperinflammation [29]. Further investigation of these trends in larger cohorts is warranted.

Several limitations must be considered when interpreting our findings. This was an observational, cross-sectional study, which precludes definitive conclusions regarding causality. The relatively small sample sizes in some subgroups limited the statistical power; although, we focused this discussion on the most consistent and larger groups. We did not have access to data on clinical outcomes such as thrombotic events or mortality, which would have allowed us to assess the predictive value of IPF.

Our findings align with the growing evidence of increased platelet activation and inflammation in COVID-19 [30]. While some studies reported an association between IPF and WBC, the specific correlations observed in our subgroup analyses need confirmation in larger, independent cohorts.

This study sheds light on the complex relationship between platelet turnover, inflammation and coagulation among COVID-19 patients. Our results suggest that IPF could serve as a biomarker for identifying patients at higher risk of inflammation and thrombosis, especially within certain subgroups. However, further research is needed to validate these findings and to clarify the mechanisms linking IPF to inflammation and thrombosis.

Clinical Implications of IPF Monitoring.

Our findings suggest several potential clinical applications for IPF measurement in COVID-19 patient management:

1. Risk Stratification at Admission: Elevated IPF levels at hospital admission, particularly when combined with elevated WBC and ANC, may identify patients at higher risk for thrombo-inflammatory complications. This could enable early implementation of intensified thromboprophylaxis protocols, particularly in younger patients (<70 years), males, and those with moderate-to-severe disease—subgroups in which we observed the strongest IPF-inflammation correlations.

2. Guiding Antithrombotic Therapy Intensity: The positive correlation between IPF and inflammatory markers in patients already receiving enoxaparin suggests that standard prophylactic dosing may be insufficient in some cases. Serial IPF monitoring could help identify patients who might benefit from intermediate-dose or therapeutic-dose anticoagulation, particularly when IPF remains elevated despite standard prophylaxis. Conversely, declining IPF levels may signal resolution of the prothrombotic state and guide the de-escalation of therapy.

3. Identification of High-Risk Comorbidity Subgroups: The strong correlation between IPF and D-dimer observed specifically in patients with congestive heart failure highlights the potential for IPF to refine risk assessment in vulnerable populations. CHF patients with elevated IPF may warrant more aggressive anticoagulation strategies and closer monitoring for thrombotic events.

4. Dynamic Monitoring Tool: Unlike static markers such as baseline D-dimer, IPF reflects real-time platelet turnover and can be measured serially without additional cost using standard hematology analyzers. This makes IPF particularly suitable for longitudinal monitoring throughout hospitalization, potentially triggering clinical interventions when values rise or remain persistently elevated.

5. Post-Discharge Surveillance: Given that thrombotic risk remains elevated up to 48 weeks after COVID-19 diagnosis, outpatient IPF monitoring could help identify patients requiring extended thromboprophylaxis after hospital discharge.

**Proposed Clinical Algorithm**

Based on our findings, we propose a preliminary risk-stratification approach:

**Low risk**: IPF within normal range, age > 70, mild disease → standard prophylactic anticoagulation.

**Intermediate risk**: Elevated IPF with moderate WBC/ANC elevation, age < 70, moderate disease → consider intermediate-dose anticoagulation and serial IPF monitoring.

**High risk**: Markedly elevated IPF with high WBC/ANC, severe disease, CHF, or elevated D-dimer → therapeutic anticoagulation with close monitoring.

This algorithm requires prospective validation in clinical trials before implementation in routine practice.

In conclusion, the consistent association between IPF and WBC suggests a common pathway connecting platelet production, systemic inflammation, and coagulation in hospitalized COVID-19 patients.

Measuring IPF is advantageous because it offers a rapid, accessible, and reproducible method for assessing thrombotic risk, not only in COVID-19 but also in other conditions with high platelet turnover or inflammation [6,30]. Monitoring IPF can provide valuable prognostic information and may help guide antithrombotic strategies in both infectious and non-infectious inflammatory states characterized by increased risk of thrombosis.

## Figures and Tables

**Table 1:** Baseline characteristics of the patients and laboratory parameters.

Demographic and Clinical Characteristics	Value
Age (years), median (range)	68 (19–101)
>70, *n* (%)	62 (47)
<70, *n* (%)	71 (53)
Weight (kg), median (range)	83.5 (50–130)
Sex, *n* (%)	133 (100)
Male	84 (63)
Female	49 (37)
Disease degree, *n* (%)	131 (100)
Mild	53 (40.5)
Moderate to severe	78 (59.5)
Active smoker, *n* (%)	13 (90)
Ischemic heart disease, *n* (%)	23 (17)
Diabetes mellitus, *n* (%)	57 (43%)
Hyperlipidemia, *n* (%)	58 (43.5%)
Chronic renal failure, *n* (%)	17 (13%)
COPD or Asthma, *n* (%)	18 (13.5%)
Chronic heart failure, *n* (%)	14 (10.5)
History of cerebrovascular accident, *n* (%)	15 (11)
Atrial fibrillation, *n* (%)	19 (14)
Solid cancer or lymphoma, *n* (%)	23 (17)
Deep vein thrombosis, *n* (%)	6 (4.5)
**Laboratory findings**	
C-reactive protein (mg/L), median (IQR)	60 (0.3–374.5)
White blood cells (*10^3^/µL), median (IQR)	7.5 (1.6–22)
Absolute neutrophil count (*10^3^/µL), median (IQR)	5.8 (0.9–19.6)
Lymphocytes (*10^3^/µL), median (IQR)	1.0 (0.1–4.1)
Fluorescence platelet count (PLT-F) (*10^3^/µL), median (IQR)	224 (78–762)
Neutrophil-to-lymphocyte ratio, median (IQR)	6.4 (0.76–74)
Platelet-to-lymphocyte ratio, median (IQR)	241.6 (64.4–1996.66)
D-dimer (mg/L), median (IQR)	0.93 (0.9–34.24)
Ferritin (ug/L), median (IQR)	675 (12–11870)
Fibrinogen (mg/dL), median (IQR)	508 (90–900)
Lactate dehydrogenase (U/L), median (IQR)	550 (248–1484)
ALT (U/L), median (IQR)	29 (8–414)
AST (U/L), median (IQR)	34 (11–332)

**Table 2:** Variable correlations of immature platelet fraction (IPF) with inflammatory and thrombotic markers in COVID-19 patients.

Subgroup	n	PLT-F	WBC	ANC	LDH	Fibrinogen	D-Dimer
Age < 70	71	N/A	r ^1^ = 0.363 *p* ^2^ = 0.026	r = 0.331 *p* = 0.033	N/A	N/A	N/A
Male	84	r = 0.295 *p* = 0.046	r = 0.290 *p* = 0.046	N/A	N/A	N/A	N/A
Disease degree 2/3	53	r = 0.342 *p* = 0.026	r = 0.326 *p* = 0.026	r = 0.293 *p* = 0.039	N/A	N/A	N/A
No ischemic heart disease	110	N/A	r = 0.292 *p* = 0.026	r = 0.25 *p* = 0.052 *n* req. ^3^ = 123	r = 0.241 *p* = 0.052 *n* req. = 133	N/A	N/A
No congestive heart failure	119	r = 0.233 *p* = 0.048	r = 0.277 *p* = 0.026	r = 0.236 *p* = 0.048	r = 0.211 *p* = 0.081 *n* req. = 174	N/A	N/A
Congestive heart failure	14	N/A	N/A	N/A	N/A	N/A	r = 0.785 *p* = 0.013
No diabetes mellitus	76	r = 0.271 *p* = 0.059 *n* req. = 105	r = 0.384 *p* = 0.013	r = 0.322 *p* = 0.033	r = 0.312 *p* = 0.035	N/A	N/A
No chronic renal failure	116	r = 0.212 *p* = 0.072 *n* req. = 172	r = 0.306 *p* = 0.013	r = 0.268 *p* = 0.026	r = 0.233 *p* = 0.061 *n* req. = 142	N/A	N/A
No hyperlipidemia	75	N/A	r = 0.403 *p* = 0.0004	r = 0.346 *p* = 0.013	N/A	N/A	N/A
No COPD or asthma	115	r = 0.249 *p* = 0.046	r = 0.258 *p* = 0.046	r = 0.228 *p* = 0.061 *n* req. = 149	N/A	N/A	N/A
Cerebrovascular accident	15	r = 0.739 *p* = 0.026	r = 0.618 *p* = 0.061 *n* req.= 18	r = 0.633 *p* = 0.061 *n* req. = 17	N/A	N/A	N/A
No hypothyroidism	122	r = 0.204 *p* = 0.078 *n* req. = 186	r = 0.28 *p* = 0.026	r = 0.242 *p* = 0.046	r = 0.219 *p* = 0.078 *n* req. = 161	N/A	N/A
No solid cancer or lymphoma	110	r = 0.271 *p* = 0.013	r = 0.345 *p* = 0.00026	r = 0.302 *p* = 0.007	r = 0.218 *p* = 0.068 *n* req. = 163	r = 0.361 *p* = 0.009	N/A
No deep vein thrombosis	127	r = 0.223 *p* = 0.052 *n* req. = 156	r = 0.270 *p* = 0.026	r = 0.230 *p* = 0.052 *n* req. = 146	N/A	N/A	N/A
Enoxaparin	77	N/A	r = 0.373 *p* = 0.007	r = 0.361 *p* = 0.007	r = 0.269 *p* = 0.078 *n* req. = 106	N/A	N/A
No plaquenil	120	r = 0.243 *p* = 0.046	r = 0.244 *p* = 0.046	N/A	N/A	N/A	N/A
Plaquenil	13	N/A	N/A	N/A	r = 0.843 *p* = 0.013	N/A	N/A
With antiplatelet therapy	41	r = 0.377 *p* = 0.065 *n* req = 53	r = 0.418 *p* = 0.046	r = 0.413 *p* = 0.046	N/A	N/A	N/A
No NOAC	120	r = 0.251 *p* = 0.026	r = 0.290 *p* = 0.013	r = 0.251 *n* = 0.026	N/A	N/A	N/A
No plasma from past COVID	109	r = 0.262 *p* = 0.039	r = 0.268 *p* = 0.039	r = 0.226 *p* = 0.078 *n* req. = 183	N/A	N/A	N/A
No ACE/ARB	79	r = 0.264 *p* = 0.062 *n* req. = 110	r = 0.425 *p* = 0.001	r = 0.393 *p* = 0.0004	r = 0.374 *p* = 0.004	N/A	N/A
No statin	79	N/A	r = 0.338 *p* = 0.026	N/A	N/A	N/A	N/A
No CCB DHP	108	r = 0.263 *p* = 0.026	r = 0.367 *p* = 0.001	r = 0.324 *p* = 0.007	r = 0.248 *p* = 0.039	N/A	N/A
No amiodarone	130	r = 0.237 *p* = 0.035	r = 0.266 *p* = 0.026	r = 0.231 *p* = 0.035	r = 0.203 *p* = 0.078 *n* req. = 188	N/A	N/A
No digoxin	131	r = 0.223 *p* = 0.048	r = 0.276 *p* = 0.013	r = 0.243 *p* = 0.033	r = 0.206 *p* = 0.068 *n* req. = 183	N/A	N/A

^1^ Pearson product–moment correlation. ^2^ Adjusted *p*-value. ^3^ Total sample size required to determine whether a correlation coefficient differs from zero. N/A not applicable, PLT-F fluorescence platelet count, WBC white blood cells, ANC absolute neutrophil count, LDH lactate dehydrogenase, COPD chronic obstructive pulmonary disease, NOAC new oral anticoagulants, ACE/ARB angiotensin converting enzyme inhibitors/angiotensin receptor blockers, CCB DHP dihydropyridine calcium channel blocker.

**Table 3:** Absolute immature platelet fraction (IPF) values in the clinical and treatment subgroups.

Subgroup	n	IPF (%)	
		Median (IQR)	Range
Total cohort	133	1.9 (0.2–25)	0.1–29
Age < 70 years	71	2.5 (0.5–27)	0.1–29
Male	84	2.3 (0.3–26)	0.1–29
Moderate-to-severe disease	78	2.7 (0.8–28)	0.1–29
Enoxaparin required	77	2.6 (0.6–27)	0.1–29
Congestive heart failure	14	3.5 (1.1–29)	0.4–29

## Data Availability

Anonymized data related to this study can be made available upon reasonable request to the corresponding author.

## Abbreviations

The following abbreviations are used in this manuscript:

IPF	Immature platelet fraction
COVID-19	Coronavirus Disease of 2019
SARS-CoV-2	Severe Acute Respiratory Syndrome Coronavirus 2
CRP	C-reactive protein
LDH	Lactate dehydrogenase
PLR	Platelet-to-lymphocyte ratio
NLR	Neutrophil-to-lymphocyte ratio
ALT	Alanine aminotransferase
AST	Asparatate aminotransferase
RP	Reticulated platelets
